# Impact of knowledge and attitude on the utilization rate of cervical cancer screening tests among Ethiopian women: A systematic review and meta-analysis

**DOI:** 10.1371/journal.pone.0239927

**Published:** 2020-12-08

**Authors:** Ayelign Mengesha Kassie, Biruk Beletew Abate, Mesfin Wudu Kassaw, Teshome Gebremeskel Aragie, Bonsa Amsalu Geleta, Wondimeneh Shibabaw Shiferaw

**Affiliations:** 1 Department of Nursing, College of Health Sciences, Woldia University, Woldia, Amhara Regional State, Ethiopia; 2 Department of Nursing, College of Health Sciences, Metu University, Metu, Oromia Regional State, Ethiopia; 3 Department of Nursing, College of Health Sciences, Debre Berhan University, Debre Berhan, Amhara Regional State, Ethiopia; University of Jos, NIGERIA

## Abstract

**Introduction:**

Cervical cancer is a major public health problem, particularly in resource-limited settings. The use of vaccination and screening tests has reduced the burden of cervical cancer in developed countries. However, the situation is quite the reverse in developing countries, including Ethiopia. Hence, this study aimed to estimate the pooled impact of knowledge and attitude on the prevalence of cervical cancer screening service utilization rates among Ethiopian women.

**Methods:**

Studies that examined cervical cancer screening service utilization among women in Ethiopia were searched from five international databases. Cochran’s Q chi-square and the I-squared test statistics were used to check the presence of heterogeneity among the included studies. The funnel plot and Egger’s regression tests were also used to assess the presence of publication bias. A weighted DerSimonian and Laird random-effects model was employed. Subgroup analysis was performed by the study population concerning the prevalence of cervical cancer screening service utilization rates. Sensitivity analysis was also conducted to assess the effect of a single study on the pooled estimates. Data analysis was performed using STATA™ Version 14 software.

**Results:**

A total of 44 studies with 28,186 study participants were included. The estimated pooled prevalence of cervical cancer screening service utilization was 8.11% (95% CI: 7.26, 8.97). After adjustment for publication bias with the trim and fill analysis, the estimated prevalence rate appeared to be 5.47% (95% CI: 4.66, 6.28). The prevalence of cervical cancer screening service utilization was higher among HIV-positive women, 16.85%, and in studies conducted among health care workers, 10.24%, than the general population. The pooled effect of knowledge on the utilization of cervical cancer screening tests among Ethiopian women was statistically significant (AOR _=_ 3.20, 95% CI: 1.63, 6.31). Similarly, the pooled estimated odds of utilizing cervical cancer screening tests were 6.1 times higher (AOR _=_ 6.09, 95% CI: 1.09, 34.36) among women who had a favorable attitude towards the screening tests.

**Conclusion:**

Knowledge and attitude had a significant impact on the prevalence of cervical cancer screening test utilization rates among women in Ethiopia. However, the prevalence of cervical cancer screening service utilization among Ethiopian women is very low. Hence, large-scale awareness programs and situation-based strategies need to be designed to increase the uptake of cervical cancer screening services in the country.

## Introduction

Globally, the incidence and mortality rates of cervical cancer have increased with 0.6 and 0.46% estimated annual rates respectively in the past 3 decades [1, 2]. The new cases of cervical cancer more often occur in all age groups of women in developing countries than in developed countries [2, 3].

Cervical cancer is the most common type of cancer and the leading cause of cancer-related deaths in women in several low and middle human development index countries [4]. Africa is hardly affected by the disease [3]. This is a source of great concern because cervical cancer is preventable and curable using the currently available methods [5, 6].

The onset of the HIV/AIDS epidemic that is highest in the region has also raised the problem of cervical cancer to a serious level [6]. In 2018, cervical cancer was the leading cause of cancer-related mortality among women in the southern, western, middle, and eastern Africa regions [7].

Cervical cancer costs the world billions of dollars [8, 9]. Cervical cancer-related costs are estimated at $407.2 per patient for outpatient services in Ethiopia and the mean inpatient cost for hospitalized patients is estimated to be $404.4 [10]. This is very huge and unimaginable for many to get treatment given the socioeconomic status of the community [10, 11].

Human papillomavirus (HPV) which is a sexually transmitted infection-causing organism is the main cause for cervical cancer [12, 13]. Currently, highly effective vaccines are available against HPV infection [14–16]. However, early detection through screening and treatment of pre-cancerous lesions remains the best possible protection against cervical cancer in resource-limited settings [17].

Human papilloma virus-based screening coupled with immediate treatment with cryotherapy has reduced both cervical cancer precursors and cervical cancer cases in many countries though HPV-based screening has been indicated to be more effective than cytology for the detection of cervical cancer precursors and prevention of cervical cancer [18].

Nevertheless, the rate of cervical cancer screening tests is reported to be low across literatures in many low and middle-income countries [19–21]. It is indicated that on average, cervical cancer screening rate is three times higher in high-income countries (63%) than in low and middle-income countries (19%) [22].

The Ethiopian cervical cancer prevention and control guidelines recommend every woman starting from the age of 30 to be screened every five years unless HIV-positive and if there is an indication starting from the age of 25 years. If a woman is HIV positive, it recommends starting screening at HIV diagnosis, regardless of age once the woman is sexually exposed [23].

In line with global guidelines, the Ethiopian cervical cancer prevention and control guidelines recommend the human papillomavirus test and cytological tests such as the use of visual inspection of the cervix with acetic acid and Pap smear tests to be used for screening depending on their availability in the health care systems nearby to the woman [23, 24].

However, the uptake of cervical cancer screening tests is unsatisfactory in Ethiopia. A study has indicated that based on the World Health Organization (WHO) non-communicable steps survey conducted in Ethiopia in 2015, the rate of cervical cancer screening tests was extremely low (2.9%) [25].

The low uptake rate of cervical cancer screening tests in many low- and middle-income countries is thought to be related to multiple barriers, including the lack of awareness and knowledge about the risk factors and the prevention methods of cervical cancer and the women's unwillingness to be tested [26].

Therefore, knowing the impact of knowledge and attitude on the utilization rate of cervical cancer screening tests is paramount in designing effective strategies. However, primary studies on the utilization rate of cervical cancer screening tests and the associated barriers in Ethiopia are inconsistent. Hence, this systematic review and meta-analysis aimed to determine the impact of knowledge and attitude on the prevalence of cervical cancer screening service utilization rates among Ethiopian women.

## Methods

### Literature search strategy

The Cochrane Library, Joanna Briggs Institute (JBI), and PROSPERO databases were searched to check whether a systematic review and meta-analysis studies exist or for the presence of ongoing project works related to the utilization of cervical cancer screening tests and the associated barriers in Ethiopia. The relevant articles were searched using PubMed, Scopus, Google Scholar, and African journals online.

For this study, relevant articles were identified using the following terms: “Cervical cancer screening”, “utilization”, “uptake”, “practice”, “factors”, “barriers” and “Ethiopia”. The key terms were used in combination using Boolean operators of “OR” or “AND”. The searches were restricted to full texts, free articles, English language publications, and human studies. This search involved articles published from January 1^st^, 2010 to April 10^th^, 2020.

Gray literature, such as surveillance reports, academic dissertations, and conference proceeding abstracts, were also examined and included when they deemed low risk. In addition, the reference lists of included articles in this systematic review and meta-analysis were used for hand searching to identify any relevant additional articles.

#### PubMed search strategy

((((((((Cervical cancer screening [MeSH Terms])) AND (Utilization)) OR (Uptake)) OR (Practice)) AND (Factors)) OR (Barriers)) AND (Ethiopia)) AND (("2010/01/01"[Date—Entry]: "2020/04/10"[Date—Entry])). Filters applied: Free full text, in the last 10 years, English, Humans.

### Article screening for eligibility and selection for inclusion

Studies were included in this systematic review and meta-analysis if they followed the following guidelines: (1) all observational study designs (cross-sectional, case-control, and cohort studies) that reported the utilization rate of cervical cancer screening tests and the associated barriers; (2 published from 2010 to 2020; (3) published in the English language; (4) abstract and, or full text available for this review; and (5) conducted in Ethiopia. Unpublished works were also considered and included when low risk was deemed. Studies were excluded if they (1) possessed a poor quality score as per the stated criteria and (2) failed to determine the desired outcomes (utilization of cervical cancer screening services and its association with knowledge and attitude of women). Furthermore, citations without abstracts and/or full texts, anonymous reports, editorials, and qualitative studies were excluded from the analysis.

### Outcomes of interest

The main outcomes of interest were the utilization rate of cervical cancer screening services and its association with the knowledge and attitude of women. The outcome variables of interest were operationally defined as follows for the study participants of the included studies in the systematic review and meta-analysis.

#### Cervical cancer screening service utilization

A woman who has been screened for the detection of any possible abnormal cells in the cervix in their lifetime. In this study, screening refers to the use of a human papillomavirus test, and/or the use of visual inspection of the cervix with acetic acid [27, 28] and, or the use of a Pap smear test [28].

#### Knowledge

Studies were included for knowledge analysis if participants’ knowledge of cervical cancer and/or about cervical cancer screening tests were reported with outcomes categorized as good and poor or other analogous binary classifications. For studies that have presented the knowledge of participants in sections, the knowledge category that covers questions about cervical cancer screening was considered. For studies that used more than 2 categories to describe the participant’s knowledge of cervical cancer and screening services, the 2 categories of good and poor were selected and included in this study. That means we selected the lowest and compared it to the highest in terms of the likelihood of attending screening tests.

#### Attitude

Similarly, for the attitude section, studies that have reported findings in the binary category as favorable and unfavorable or positive and negative were included.

### Data extraction

The authors have developed a data extraction platform on the Excel sheet that consists of the name of authors, year of study, year of publication, study design, study setting, participant type, sample size, prevalence rate of cervical cancer screening service utilization, participants’ knowledge and attitude towards cervical cancer and screening tests. The data extraction sheet was piloted using 6 papers randomly. Then, the extraction form was adjusted after having the piloted template. Two of the authors independently extracted all the necessary data from each article using the data extraction sheet. The third author checked the correctness of the data independently. Any disagreements between authors who extracted the data were resolved through discussions with the third reviewer and fourth reviewer when required. For articles that did not provide details of their study background, corresponding authors were contacted through e-mail and asked for relevant information, such as study time, region, or others.

### Quality assessment

Two independent authors assessed the methodological quality of all of the potential studies to be included in the analysis. Any failure to reach an agreement between the authors on whether to include or exclude studies was resolved through discussion or, if consensus could not be reached, consultation with a third independent author was considered. The quality of each included study was assessed using the JBI quality appraisal checklist for case-control and cross-sectional studies because the articles included in this study were case-control and cross-sectional in design [29].

The JBI quality appraisal checklist has several key criteria to appraise case-control studies, including [1] group comparability other than the presence of disease in the cases or the absence of disease in the controls [2], matching appropriateness of cases and controls [3], similarity of criteria used for identification of cases and controls [4], exposure measurement in a standard, valid and reliable way [5], similarity of exposure measurement for cases and controls [6], identification of confounding factors [7], clarity of strategies to deal with confounding factors [8], outcome assessment in a standard, valid and reliable way for cases and controls [9], meaningfulness of period of exposure and [10] appropriateness of statistical methods used.

The items in the checklist used to appraise cross-sectional studies were [1] clarity of inclusion criteria definitions [2], extent of study participant and setting description [3], valid and reliable exposure measurement [4], presence of clear objective, and use of standard criteria for measurement of the condition of interest [5], identification of confounding factors [6], clarity of strategies to handle confounding factors [7], valid and reliable way of outcome measurement and [8] appropriateness of statistical methods used [29]. Studies were considered low risk when scored 50% and above of the JBI quality assessment indicators.

### Statistical analysis

To obtain the pooled estimated impact of knowledge and attitude on the prevalence of cervical cancer screening service utilization rates among Ethiopian women, a meta-analysis using the random-effects model was performed due to the presence of heterogeneity [30].

Cochran’s Q chi-square statistic and the I^2^ tests were run to assess the random variations between primary studies [31]. In this study, heterogeneity was interpreted as an I^2^ value of 0% = no heterogeneity, 25%-50% = low, 50%-75% = moderate, and ≥ 75% = high [31]. In the case of high heterogeneity, subgroup and sensitivity analyses were run to identify possible moderators for the heterogeneity.

Potential publication bias was assessed by visually inspecting funnel plots and objectively using Egger’s bias test [32]. The trim and fill analysis was performed to assess for any publication bias based on the assumption that the effect sizes of all the studies are normally distributed around the center of a funnel plot in the absence of publication bias. The meta-analysis was performed using STATA™ Version 14 software [33]. Finally, for all analyses, a p-value of less than 0.05 was considered to declare statistically significant values.

### Presentation and reporting of results

The results of this systematic review and meta-analysis were reported based on the Preferred Reporting Items for Systematic Review and Meta-Analysis statement (PRISMA) guidelines [34]. The entire process of study screening, selection, and inclusion are shown with the support of a flow diagram. Moreover, tables and narrative summaries are used to report the risk of bias for every eligible study.

## Results

### Search results

**Fig 1:**
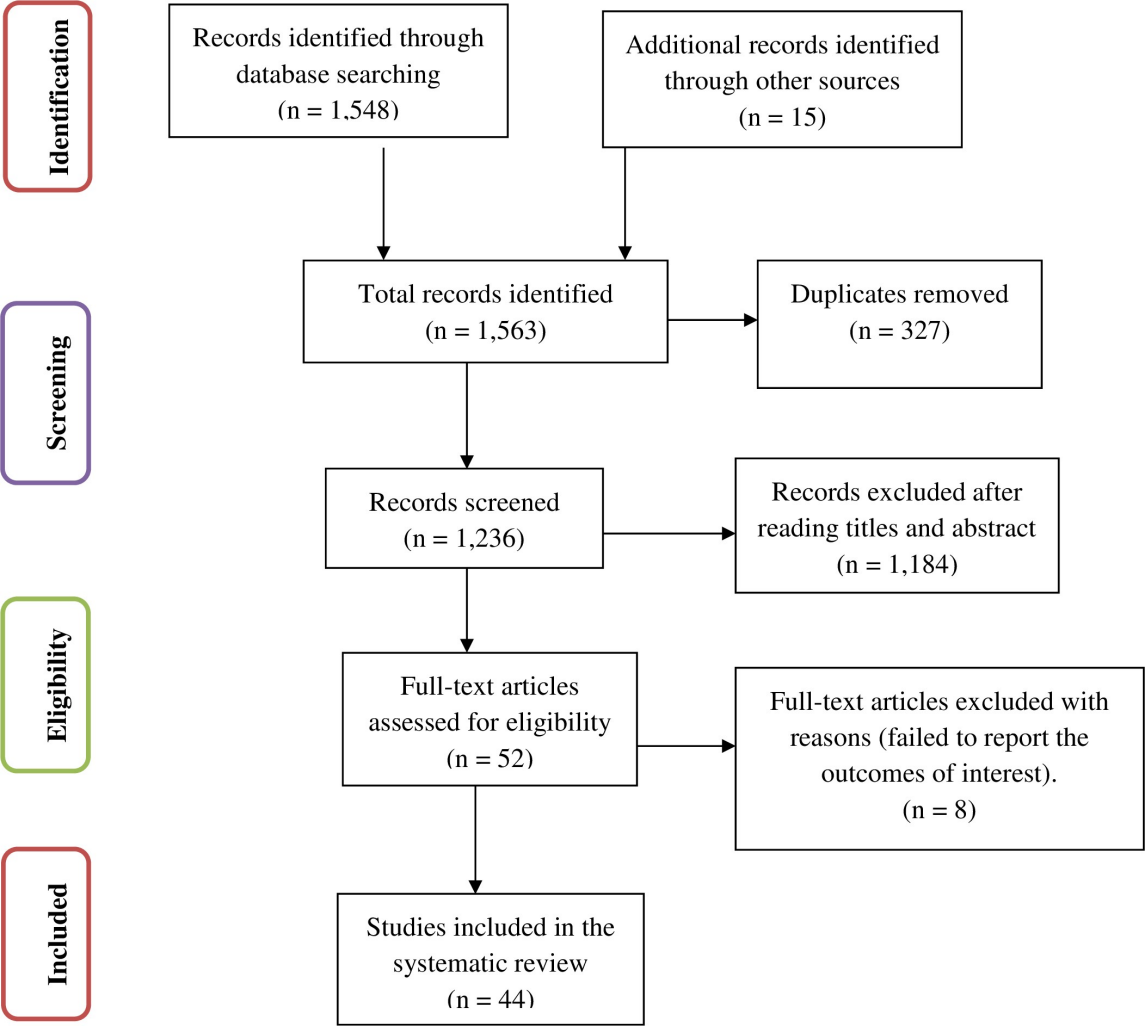
PRISMA flow chart of study selection for the prevalence of cervical cancer screening service utilization in Ethiopia.

In the first step of article searches, 1,548 studies that were published from 2010–2020 were retrieved from four international databases. Fifteen studies (n = 15) were retrieved through manual searches from grey literature. Of the 1,563 retrieved studies, 327 duplicate records were identified, and after duplication removal, a total of 1,236 articles remained for screening. Then, 1,184 studies were excluded after reading their title and abstracts because either these studies were not conducted in Ethiopia or they were not in line with the objective of this systematic review and meta-analysis. Finally, 52 studies were screened for full-text review, and 44 studies with 28,186 study participants were included. The remaining 8 articles were excluded because they failed to report the outcomes of interest (prevalence of cervical cancer screening tests or impact of knowledge and attitude on the utilization rate of cervical cancer screening tests). The detailed steps in the screening process are shown in the PRISMA flow chart below (Fig 1).

### Baseline characteristics of the included studies

In this systematic review and meta-analysis, a total of 44 studies with 28,186 study participants were included to estimate the pooled impact of knowledge and attitude on the prevalence of cervical cancer screening service utilization rates among Ethiopian women.

Among the included studies, 42 had reported the prevalence of cervical cancer screening service utilization, 24 had reported the level of association between participants’ knowledge and cervical cancer screening service utilization, and 12 studies had also reported the effect of attitude on the utilization rate of cervical cancer screening services.

Concerning the study design, 42 of the studies included were cross-sectional, and the remaining 2 were case-control in design. The studies included in this systematic review and meta-analysis varied substantially in sample size, ranging from 202 to 5,823.

Overall information regarding the prevalence of cervical cancer screening service utilization was obtained from various regions of the country. Ten of the studies involved participants from the Amhara region [35–44], eleven from the Oromia region [45–55], nine from the SNNPR [56–64], four from Tigrai [65–68], eight from Addis Ababa [69–76], one study involving participants from the Amhara, SNNPR and Afar regions [77], and another study involving participants countrywide [25]. Regarding sampling, all of the studies used the probability sampling technique (**Table 1**).

**Table 1:** Baseline characteristics of included studies to analyze the impact of knowledge and attitude on the utilization rate of cervical cancer screening tests in Ethiopia.

Author name	Publication year	Region	Setting	Design	Participant type	Sample Size	Prevalence of Screening	Standard error of screening	Adjusted odds ratio knowledge	Adjusted odds ratio attitude	Quality assessment
Abamech F, et al.	2019	SNNPR	Institution-based	Cross-sectional	General	402	9.2	1.44			9
Abdulkadir IR, et al.	Not published	Addis Ababa	Institution-based	Cross-sectional	Students	387	5.2	1.13			7
Andargie A, et al.	2015	Amhara	Institution-based	Cross-sectional	General	603	8.3	1.12			8
Ashagrie A, et al.	Not published	Oromia	Institution-based	Cross-sectional	HIV Positive	311	16	2.08	5		9
Assefa AA, et al.	2019	SNNPR	Institution-based	Cross-sectional	HIV Positive	342	40.1	2.65	1.7	3.7	9
Aweke YH, et al.	2017	SNNPR	Community-based	Cross-sectional	General	*583*	9.9	1.24			7
Aynalem BY, et al.	2020	Amhara	Community-based	Cross-sectional	General	822	5.4	0.79	4.02	3.23	9
Bante SA, et al.	2019	Amhara	Community-based	Cross-sectional	General	517	20.9	1.79	0.59	3.38	8
Bayu H, et al.	2016	Tigrai	Community-based	Cross-sectional	General	1186	19.8	1.16	2.36		8
Belete N, et al.	2015	Addis Ababa	Institution-based	Cross-sectional	HIV Positive	322	11.5	1.78			9
Berhanu T, et al.	2019	Addis Ababa	Community-based	Cross-sectional	Health workers	291	9.3	1.70	1.31	1.13	9
Bulto GA, et al.	2019	Oromia	Institution-based	Cross-sectional	HIV Positive	423	2.1	0.70			7
Chaka B, et al.	2018	More than one	Institution-based	Cross-sectional	General	799	3.3	0.63			7
Deresse M, et al.	2018	SNNPR	Community-based	Cross-sectional	General	821	15.1	1.25			9
Dirriba AB, et al.	2019	Oromia	Institution-based	Cross-sectional	General	320	29.9	2.56			9
Dulla D, et al.	2017	SNNPR	Institution-based	Cross-sectional	Health workers	367	11.4	1.66	0.52		8
Emru K, et al.	Not published	Addis Ababa	Institution-based	Cross-sectional	HIV Positive	411	25.5	2.15			9
Erku DA, et al.	2018	Amhara	Institution-based	Cross-sectional	HIV Positive	302	23.5	2.44	3.02		9
Gamshe EN, et al.	2019	Addis Ababa	Institution-based	Cross-sectional	Students	202	12.9	2.36	10.3		7
Gebreegziabher M, et al.	2016	Tigrai	Institution-based	Cross-sectional	Health workers	225	10.7	2.06	2.4	3.42	8
Gebregziabher D, et al.	2019	Tigrai	Institution-based	Cross-sectional	Students	344	17.2	2.03			8
Gebru Z, et al.	2016	SNNPR	Community-based	Cross-sectional	General	643	5.9	0.93			9
Gelibo T, et al.	2017	National	Community-based	Cross-sectional	General	5823	2.9	0.22			8
Geremew AB, et al.	2018	Amhara	Community-based	Cross-sectional	General	1137	3	0.51	3.7	9	8
Getachew E, et al.	Not published	Addis Ababa	Institution-based	Cross-sectional	General	520	3.5	0.81	5.6		7
Getahun T, et al.	2020	Amhara	Community based	Cross-sectional	General	821	2.9	0.59			9
Haile EL, et al.	2018	Oromia	Institution-based	Cross-sectional	General	412	3.2	0.87			7
Heyi WD, et al.	2018	Oromia	Community-based	Cross-sectional	General	845	5.8	0.80	6.95		8
Kasa AS, et al.	2018	Amhara	Community-based	Cross-sectional	General	735	7.3	0.96			9
Kassa RT, et al.	2019	Oromia	Community-based	Cross-sectional	General	390	16.1	1.86	1.2	1.8	9
Michael E, et al.	Not published	Oromia	Community-based	Cross-sectional	General	250	17.6	2.41	3.59	3.86	8
Mulatu K, et al.	2017	SNNPR	Institution-based	Cross-sectional	Students	209	14.8	2.46			9
Muluneh BA, et al.	2019	Amhara	Institution-based	Case-control	Sex workers	230			0.99	2.62	7
Nega AD, et al.	2019	Amhara	Institution-based	Cross-sectional	HIV Positive	460	10	1.40			8
Nigussie T, et al.	2019	Oromia	Community-based	Cross-sectional	General	737	15.5	1.33	3.47		8
Seyoum T, et al.	2017	SNNPR	Institution-based	Cross-sectional	Health workers	281	9.6	1.76	1.78		8
Shiferaw S, et al.	2018	Addis Ababa	Institution-based	Cross-sectional	HIV Positive	581	10.8	1.29			9
Solomon K, et al.	2019	Oromia	Institution-based	Cross-sectional	HIV Positive	475	24.8	1.98			9
Tadesse A, et al.	Not published	Oromia	Institution-based	Cross-sectional	Students	667	2.2	0.57			8
Teame H, et al.	2019	Tigrai	Institution-based	Case-control	General	624			2.42	15.1	7
Tefera F, et al.	2017	Amhara	Community-based	Cross-sectional	General	620	11	1.26	11.1		8
Tekle T, et al.	2020	SNNPR	Institution-based	Cross-sectional	General	516	22.9	1.85	0.78		9
Tilahun T et al.	2019	Oromia	Institution-based	Cross-sectional	Students	805	0.5	0.25			7
Woldetsadik AB, et al.	2020	Addis Ababa	Institution-based	Cross-sectional	General	425	12.2	1.59	2.48		8

### The pooled estimate of cervical cancer screening service utilization in Ethiopia

The prevalence of cervical cancer screening service utilization reported in the included studies ranges from 0% [53] to 40.1% [56]. For the study with zero prevalence, a continuity correction formula that is 0.5 was considered [53].

The possible reason for the zero prevalence might be due to differences in the setting because the study conducted by Tilahun T. *et al*. was conducted among university students who are usually young aged [53]. Therefore, the result might be due to the age of participants because screening is recommended in Ethiopia starting from the age of 30 years unless there is another clear indication [23].

**Fig 2:**
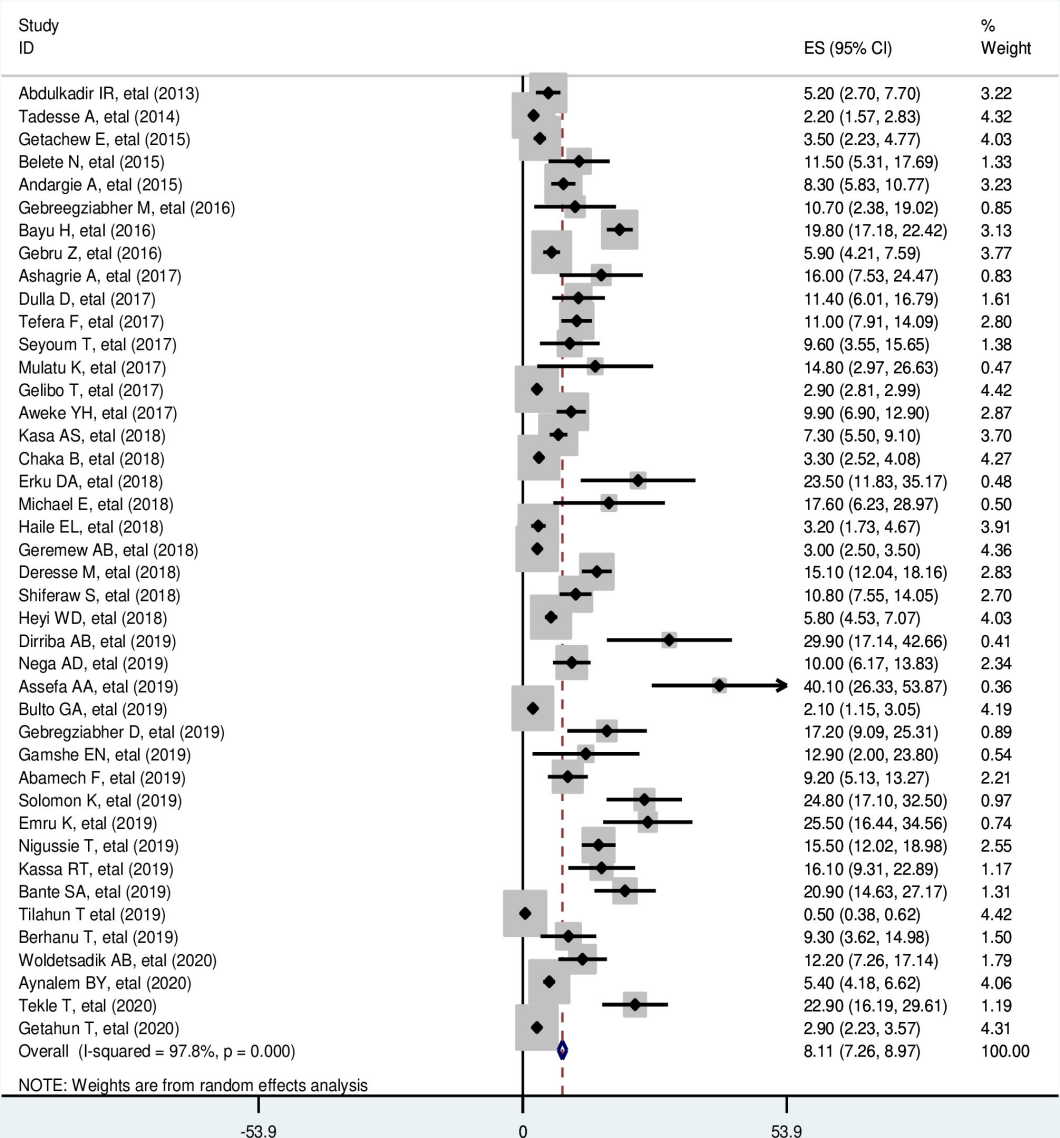
Forest plot for the prevalence of cervical cancer screening service utilization.

In this meta-analysis, the random-effects model revealed that the estimated pooled prevalence of cervical cancer screening service utilization in Ethiopia was 8.11% (95% CI: 7.26, 8.97). There was a significant level of heterogeneity among the included studies (I^2^ = 97.8%; p ≤ 0.001) (Fig 2).

### Subgroup analysis

Subgroup analysis was performed through stratification using the study population to identify the sources of heterogeneity for the pooled prevalence of cervical cancer screening service utilization rate in Ethiopia.

**Fig 3:**
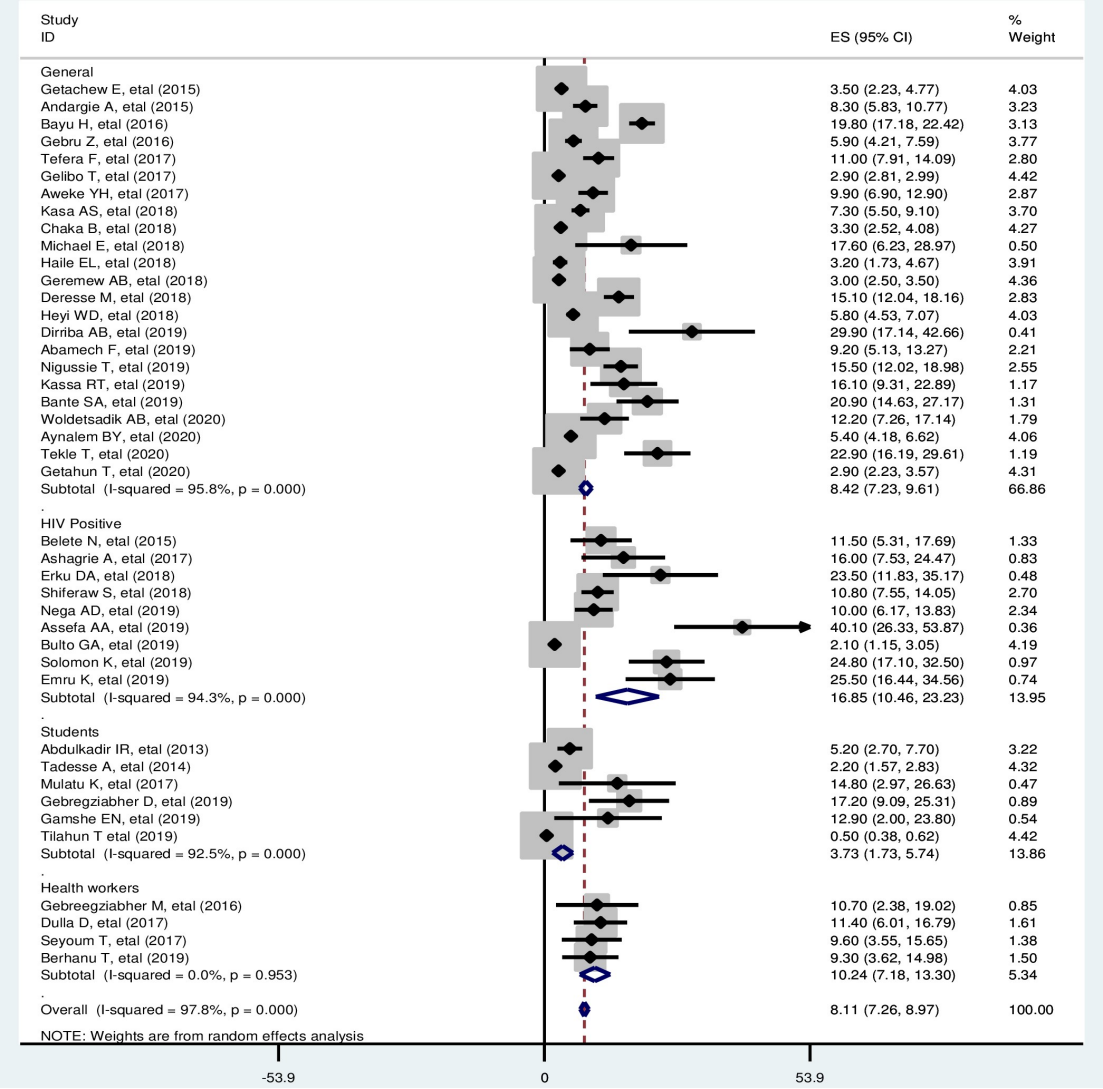
Subgroup analysis by the study population.

In the subgroup analysis, the prevalence of cervical cancer screening service utilization was found to be 16.85% in studies conducted among HIV-positive women, 10.24% in studies conducted among health care workers, 8.42% in the general population of women, and 3.73% in studies conducted among college or university students (Fig 3).

### Sensitivity analysis

**Fig 4:**
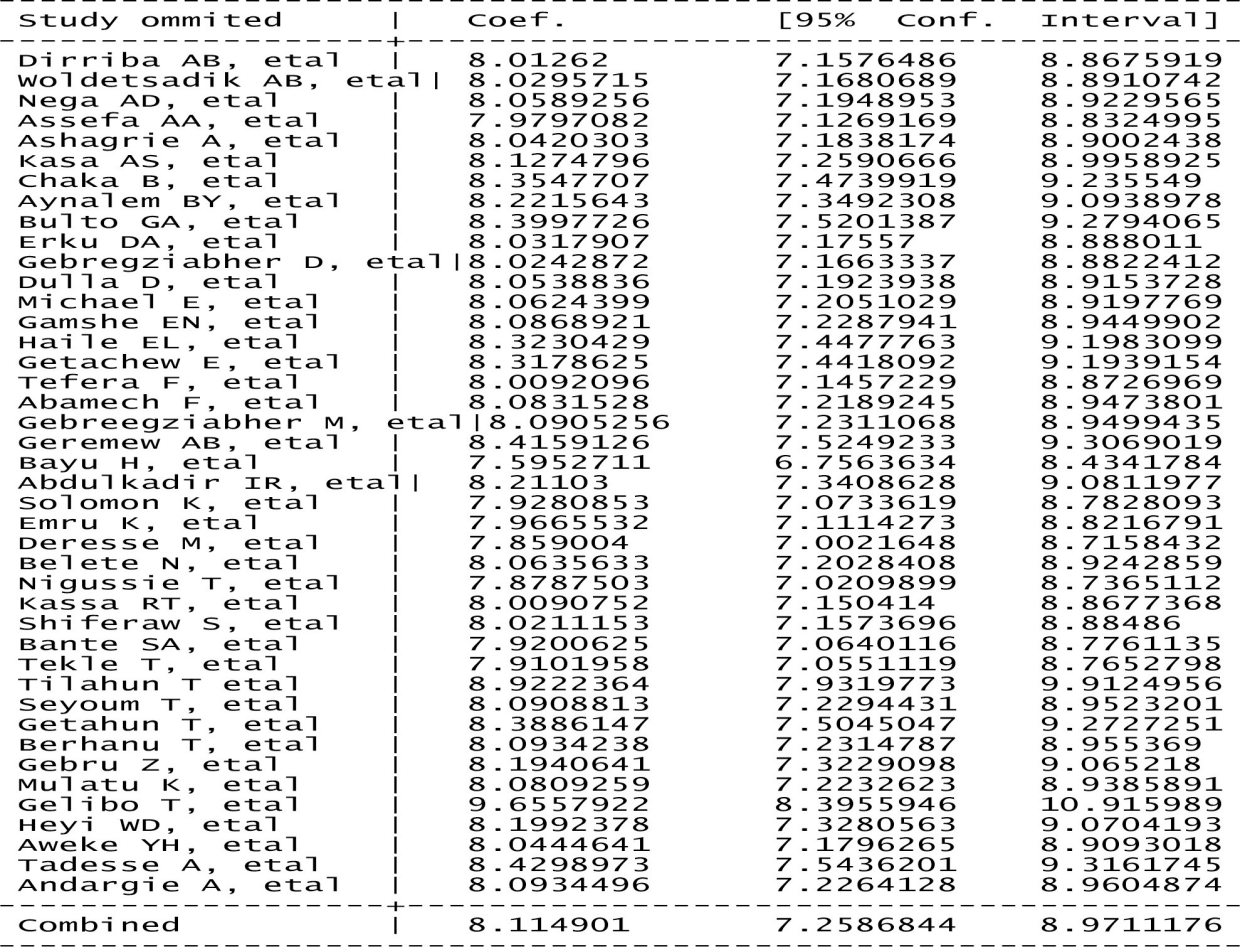
Results of the sensitivity analysis of the 42 studies in the meta-analysis.

To evaluate the effect of individual studies on the pooled prevalence of cervical cancer screening service utilization in Ethiopia, a sensitivity analysis was performed using the random-effects model. The results from the sensitivity analysis revealed that a study conducted by Gelibo T. *et al*. influenced the pooled estimated prevalence of cervical cancer screening service utilization rates among Ethiopian women. The exclusion of the Gelibo et al. study increased the overall rate from 8.11% (7.26, 8.97) to 9.66 (8.40, 10.92). This might be due to the large number of participants included in the Gelibo T. *et al*. study because unlike the other studies, the Gelibo T. *et al*. study was conducted on 5,823 study participants and involved participants from the entire country [25] (Fig 4).

### Publication bias test

There was a publication bias among the studies included to estimate the pooled prevalence of cervical cancer screening service utilization among Ethiopian women, as illustrated by the asymmetrical distribution of funnel plot tests (S1 Fig). Similarly, the result of Egger’s tests for the funnel plot was statistically significant for the presence of publication bias (P ≤ 0.001).

### Trim and fill analysis

**Fig 5:**
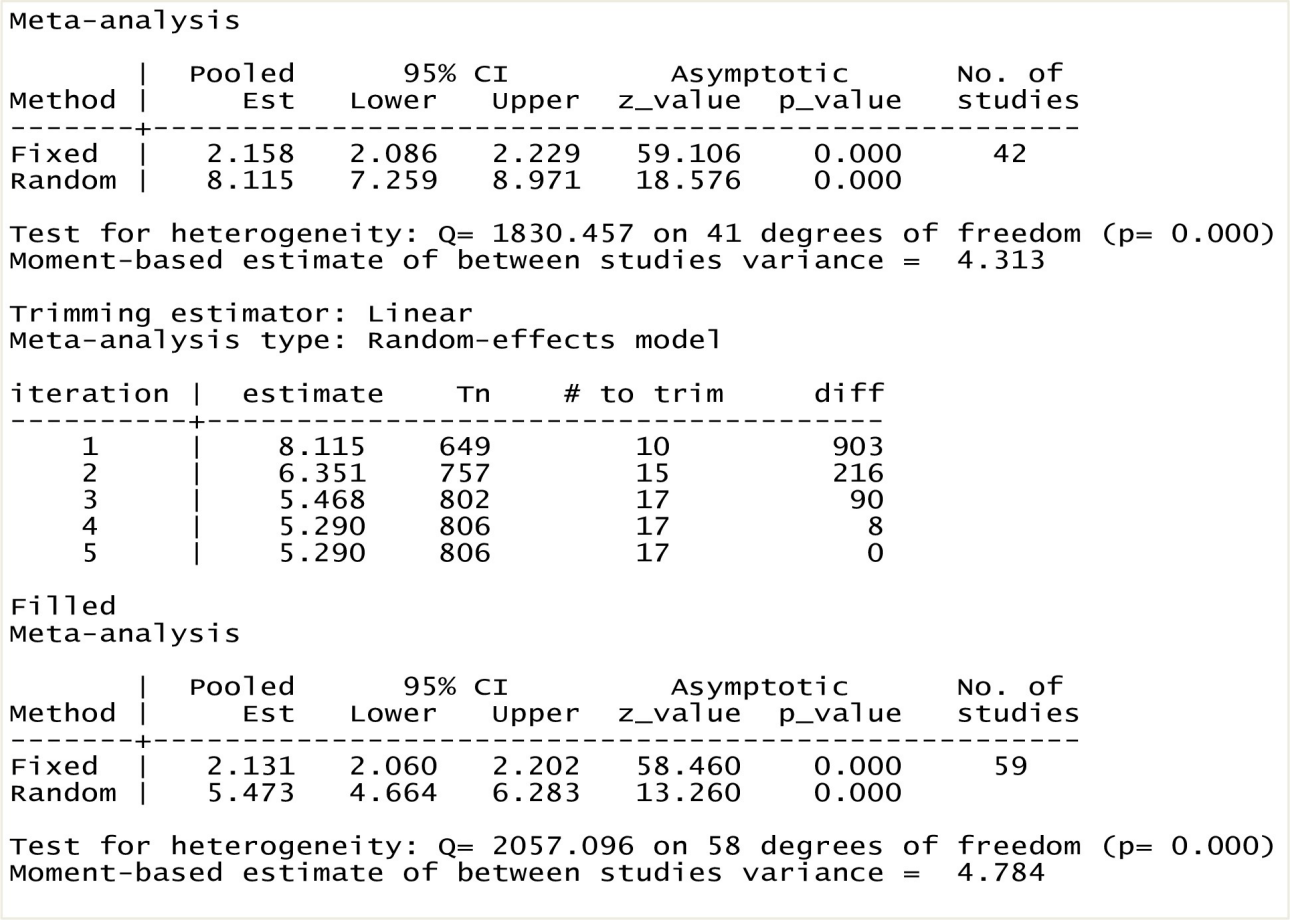
Trim and fill analysis for the prevalence of cervical cancer screening service utilization.

A trim and fill analysis was performed to estimate the number of missing studies that might exist, thereby reducing and adjusting publication bias in the included studies. After adjustment for publication bias, the estimated pooled prevalence of cervical cancer screening service utilization among Ethiopian women was 5.47 (95% CI: 4.66, 6.28), with a very low level of heterogeneity among the studies (I^2^_=_ 4.31%; p ≤ 0.001) (Fig 5).

### The effect of knowledge on utilization of cervical cancer screening services

**Fig 6:**
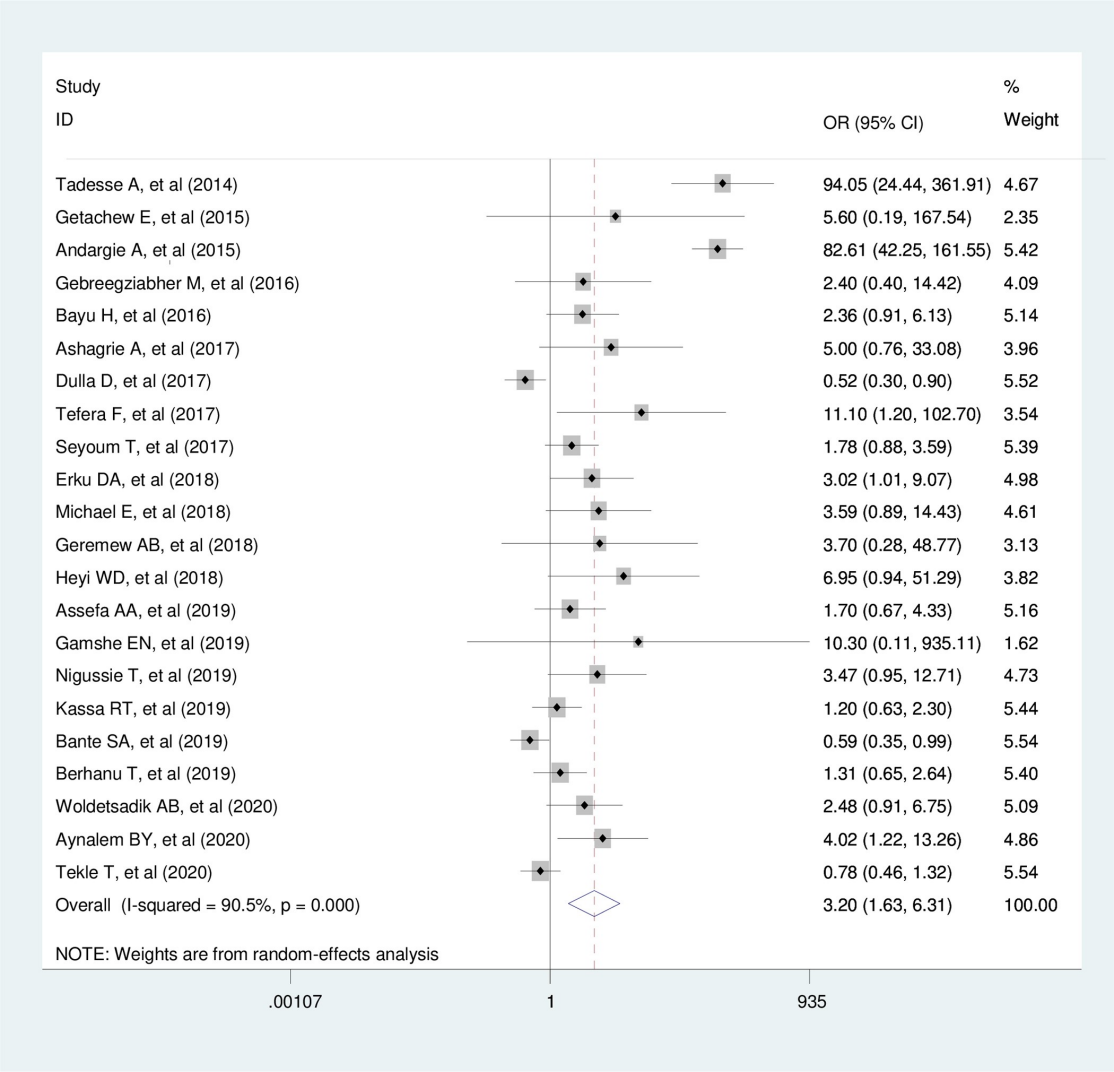
The pooled effect of knowledge on cervical cancer screening service utilization. Egger’s regression test also showed that there was no publication bias for the studies included to estimate the pooled effect of knowledge on cervical cancer screening service utilization among women (P = 0.062).

According to this meta-analysis, the pooled estimated effect of knowledge on the utilization of cervical cancer screening services in Ethiopian women was statistically significant. The odds of utilizing cervical cancer screening tests were 3.2 times higher among women who had good knowledge regarding cervical cancer and its screening tests than those who had poor knowledge (AOR _=_ 3.20, 95% CI: 1.63, 6.31, I ^2^_=_ 90.5% with p ≤ 0.001) (Fig 6).

### The effect of attitude on the utilization of cervical cancer screening services

**Fig 7:**
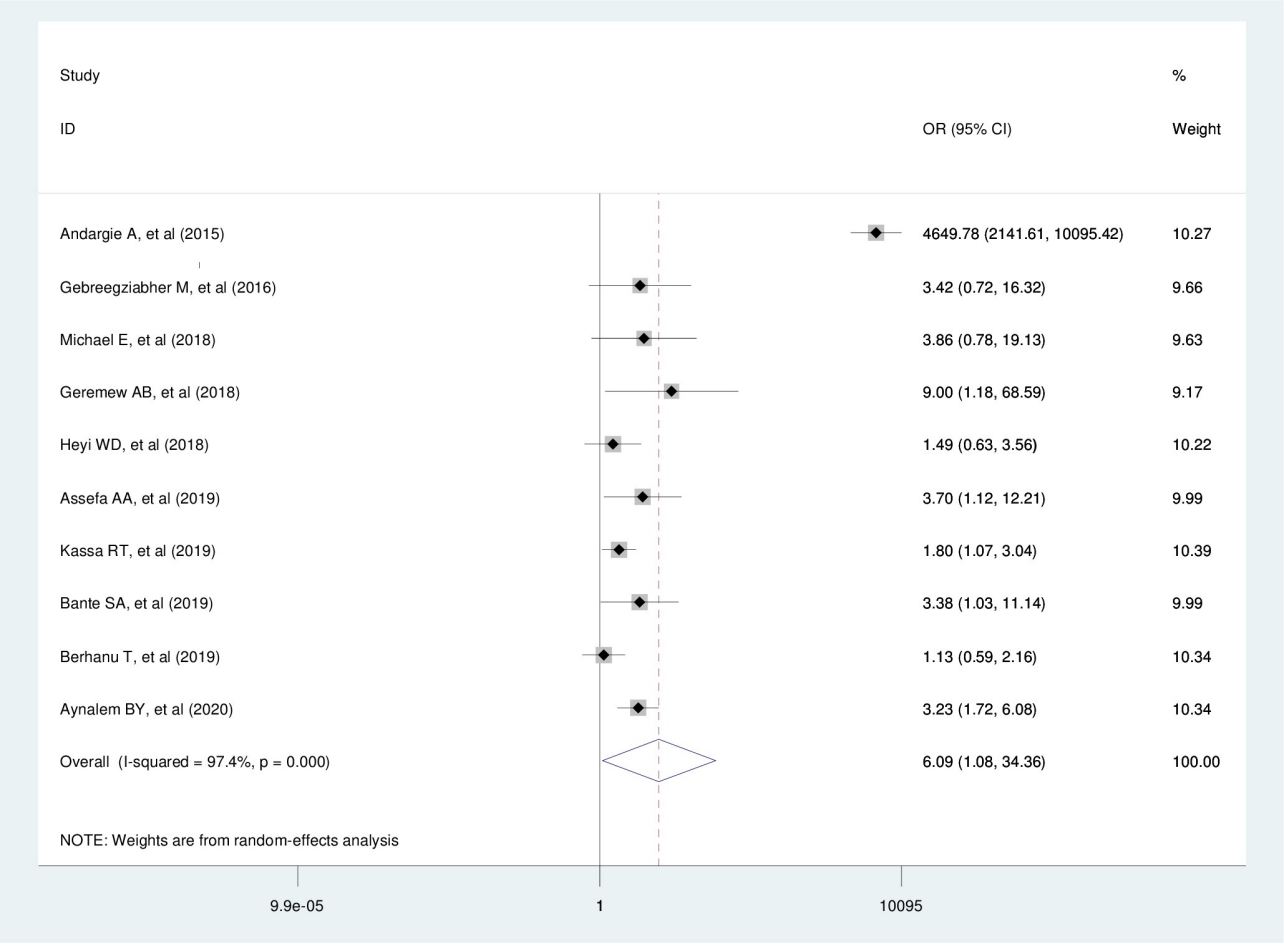
The pooled effect of attitude on cervical cancer screening service utilization.

Ethiopian women with a favorable attitude towards the utilization of cervical cancer screening tests were 6.1 times more likely than those with poor attitudes to have a test (AOR = 6.09, 95% CI: 1.09, 34.36, I^2^ = 97.4%, p ≤ 0.001). However, a very high odds ratio of 46.49 was observed under the Andargie A, et al. study row. This could be due to the difference in the reporting of odds ratios in the original studies. Unlike other studies that have reported adjusted odds ratios, the study conducted by Andargie A et al. did not report the adjusted odds ratio on the effect of attitude [44]. As a result, the crude odds ratio was used in the case of Andargie A et al.’s study (Fig 7).

In the publication bias assessment, Egger’s regression test indicated that there was no publication bias for the studies included to estimate the pooled effect of attitude on cervical cancer screening service utilization (P = 0.776).

## Discussion

The World Health Organization recommends cervical cancer screening tests to be included as part of well-planned and implemented screening programs in every country's health care policy [78]. However, the uptake of cervical cancer screening services in Ethiopia is not well established. Hence, this systematic review and meta-analysis were conducted to estimate the pooled effect of knowledge and attitude on the prevalence of cervical cancer screening service utilization rates among Ethiopian women.

In this study, the estimated pooled prevalence of cervical cancer screening service utilization among Ethiopian women was 8.1%. However, after adjustment with the trim and fill analysis for publication bias, the estimated pooled prevalence of cervical cancer screening service utilization rate appeared to be 5.5%. This finding is higher than a previously estimated rate of screening in Ethiopia, which is 0.8 [79]. It is also higher than another study conducted based on the population-based WHO Steps survey that the utilization rate of cervical cancer screening services in Ethiopia was found to be 2.9% [25]. This suggests that the uptake of cervical cancer screening rates is increasing in Ethiopia though still too low. A consistent finding has been reported in Nigeria (8.0%) [80].

However, the result is lower than a finding from European countries that the coverage of cervical cancer screening tests ranges from 10% to 79% [81]. Consistent with the European report, a study in Minnesota, United States of America, showed that 60.8% of women had received Pap-HPV co-test screening with a significant increment among women aged 30–65 years, from 10.0% in 2007 to 60.8% in 2016 [82]. It is also lower than other findings across the literature: Kenya, 16.4% [83], India, 29.8% [84], South Africa, 52% [85], and China, 20.7% [86].

These discrepancies might be due to differences in the developmental level of the countries. Unlike developed countries, the availability of cervical cancer screening programs is limited in developing countries [87, 88]. In addition, cervical cancer screening services are opportunity-based in many low- and middle-income countries [19]. This can be justified by the lack of adequate infrastructure and the availability of many other public health problems that need prioritization [89]. It might also be due to differences in the educational level and the knowledge of women in these countries because education is one of the major tools and has an immense contribution to increasing the uptake of cervical cancer screening tests [90, 91].

In the subgroup analysis, the prevalence of cervical cancer screening service utilization was higher among HIV-positive women (16.85%) and in studies conducted among health care workers (10.24%) than in the general population. This could be due to differences in the knowledge of participants. It is obvious that health care workers will have better knowledge and information on cervical cancer screening services. In addition, people with HIV are not only closer to healthcare-related information but also at a higher risk of cervical cancer and might obtain better opportunities and recommendations from their health care providers [92]. Furthermore, the Ethiopian cervical cancer prevention and control guidelines recommend that if a woman is HIV positive, screening should be started at HIV diagnosis regardless of age once the woman is sexually exposed [23].

A consistent finding has been reported that in South Africa, 25.3% of the women who attended a health care facility were screened for cervical cancer [93]. Similarly, higher screening rates were seen in a more educated woman (25.2%) in the Kenyan study [83]. The possible reason could be that people who attend health care facilities have a higher chance of getting information from their health care workers and other sources. Likewise, women who have higher educational levels might obtain information from different sources and might have a better understanding of the benefits of using cervical cancer screening tests [90, 91, 94].

The pooled odds of being screened among women who have better knowledge about cervical cancer and its screening services was 3.2 times higher than those who have poor knowledge. Similarly, Ethiopian women who had a favorable attitude towards the utilization of cervical cancer screening tests were 6.1 times more likely to have a test than those with poor attitude. This is encouraging. However, various studies in different parts of Ethiopia have shown that the majority of women not only have insufficient knowledge about cervical cancer screening tests but many of them also do not have information about the disease itself [64, 95, 96]. This suggests that awareness programs need to be implemented strategically on a regular basis.

### Strength and limitations

This meta-analysis has its strengths because it has used a pre-specified protocol for search strategy and data abstraction and used internationally accepted tools for a critical appraisal system for the quality assessment of individual studies.

As a limitation, it is difficult to determine if the results from various regions are representative of the entire country because the majority of the data were found from four (Oromia, Amhara, SNNPR, Tigray) of the 9 regions and the country's capital city Addis Ababa. Only one study was found involving participants from all regions of the country.

Furthermore, all the included studies in this systematic review and meta-analysis were either institution-based or were conducted in urban settings. Hence, the results of this study may not represent rural women in Ethiopia.

## Conclusion

Knowledge and attitude had a significant impact on the prevalence of cervical cancer screening test utilization rates among Ethiopian women. However, the prevalence of cervical cancer screening service utilization among Ethiopian women is very low. Hence, large-scale awareness creation programs and situation-based strategies need to be designed to increase the uptake of cervical cancer screening services in the country. Furthermore, addressing women living in rural areas is highly recommended, as no sufficient evidence has been found on their involvement in screening programs.

## Supporting information

S1 Fig Funnel plot test for publication bias in cervical cancer screening service utilization. (DOCX)

S1 Table (PDF)

## Abbreviations

AOR: Adjusted Odds Ratio
CI: Confidence Interval
HPV: Human Papilloma Virus
JBI: Joanna Briggs Institute
SNNPR: Southern Nations, Nationalities, and Peoples Region
PRISMA: Preferred Reporting Items for Systematic Reviews and Meta-Analyses
