# Supplementary material for: Impact of knowledge and attitude on the utilization rate of cervical cancer screening tests among Ethiopian women: A systematic review and meta-analysis
Source: PLoS One. 2020 Dec 8;15(12):e0239927. doi: 10.1371/journal.pone.0239927 (PMC7723289; doi:10.1371/journal.pone.0239927)
Supplement: S1 Fig — (DOCX) [file pone.0239927.s001.docx]

**S1 Fig:** Funnel plot test for publication bias in cervical cancer screening service utilization.
