# Supplementary material for: Impact of knowledge and attitude on the utilization rate of cervical cancer screening tests among Ethiopian women: A systematic review and meta-analysis
Source: PLoS One. 2020 Dec 8;15(12):e0239927. doi: 10.1371/journal.pone.0239927 (PMC7723289; doi:10.1371/journal.pone.0239927)
Supplement: S1 Table — (PDF) [file pone.0239927.s002.pdf]

| Study | Author | Yearofstudy | Publicationyear | Region | Setting | Design | Participanttype | SampleSize | Classofsamplesize | Pscreening | Sescreening | Cut of point | AORKwlge | LBCIKwlge | UBCIKwlge | Logorknwedge | Selogorknwledge | AORattitude | LBCIAttitude | UBCIattitude | Logorattitude | Selogorattitude | |
|---|---|---|---|---|---|---|---|---|---|---|---|---|---|---|---|---|---|---|---|---|---|---|---|
| 1 | Dirriba AB, etal | 2018 | 2019 | Oromia | Institution based | Cross-sectional | General | 320 | <384 | 29.9 | 2.559290673 | | | | | | | | | | | | |
| 2 | Woldetsadik AB, etal | 2017 | 2020 | Addis Ababa | Institution based | Cross-sectional | General | 425 | >384 | 12.2 | 1.587569359 | Not specified | 2.48 | 1.18 | 5.19 | 0.90825856 | 0.510841439 | | | | | | |
| 3 | Nega AD, etal | 2016 | 2019 | Amhara | Institution based | Cross-sectional | HIV Positive | 460 | >384 | 10 | 1.398757212 | | | | | | 0 | | | | | | |
| 4 | Assefa AA, etal | 2019 | 2019 | SNNPR | Institution based | Cross-sectional | HIV Positive | 342 | <384 | 40.1 | 2.650162745 | Mean | 1.7 | 0.7 | 4.2 | 0.530628251 | 0.477252218 | 3.7 | 1.8 | 7.5 | 1.30833282 | 0.609047775 | |
| 5 | Ashagrie A, etal | 2017 | Not published | Oromia | Institution based | Cross-sectional | HIV Positive | 311 | <384 | 16 | 2.078832222 | Mean | 5 | 1.6 | 15.88 | 1.609437912 | 0.964002226 | | | | | 0 | |
| 6 | Kasa AS, etal | 2017 | 2018 | Amhara | Community based | Cross-sectional | General | 735 | >384 | 7.3 | 0.959527945 | | | | | | 0 | | | | | 0 | |
| 7 | Muluneh BA, etal | 2016 | 2019 | Amhara | Institution based | Case-control | Sex workers | 230 | <384 | | | Mean | 0.99 | 0.4 | 2.44 | -0.010050336 | 0.364358593 | 2.62 | 0.93 | 7.33 | 0.963174318 | 0.645362788 | |
| 8 | Chaka B, etal | 2015 | 2018 | More than one | Institution based | Cross-sectional | General | 799 | >384 | 3.3 | 0.631970516 | | | | | | 0 | | | | | 0 | |
| 9 | Aynalem BY, etal | 2018 | 2020 | Amhara | Community based | Cross-sectional | General | 822 | >384 | 5.4 | 0.788327251 | Mean | 4.02 | 2.07 | 7.77 | 1.391281903 | 0.609047775 | 3.23 | 2.52 | 4.12 | 1.172482137 | 0.322681394 | |
| 10 | Bulto GA, etal | 2016 | 2019 | Oromia | Institution based | Cross-sectional | HIV Positive | 423 | >384 | 2.1 | 0.697157349 | | | | | | 0 | | | | | 0 | |
| 11 | Erku DA, etal | 2017 | 2018 | Amhara | Institution based | Cross-sectional | HIV Positive | 302 | <384 | 23.5 | 2.439839046 | Mean | 3.02 | 2.31 | 7.15 | 1.105256831 | 0.56122449 | | | | | 0 | |
| 12 | Gebregziabher D, etal | 2018 | 2019 | Tigrai | Institution based | Cross-sectional | Students | 344 | <384 | 17.2 | 2.034698995 | | | | | | 0 | | | | | 0 | |
| 13 | Dulla D, etal | 2015 | 2017 | SNNPR | Institution based | Cross-sectional | Health workers | 367 | <384 | 11.4 | 1.658961298 | Mean | 0.52 | 0.19 | 1.37 | -0.653926467 | 0.277111747 | | | | | 0 | |
| 14 | Michael E, etal | 2017 | Not published | Oromia | Community based | Cross-sectional | General | 250 | <384 | 17.6 | 2.408518217 | Mean | 3.59 | 1.4 | 9.14 | 1.278152203 | 0.709715701 | 3.86 | 1.29 | 11.54 | 1.350667183 | 0.81672503 | |
| 15 | Gamshe EN, etal | 2012 | 2019 | Addis Ababa | Institution based | Cross-sectional | Students | 202 | <384 | 12.9 | 2.35845855 | | 10.3 | 1.29 | 82.6 | 2.332143895 | 2.300307596 | | | | | 0 | |
| 16 | Haile EL, etal | 2017 | 2018 | Oromia | Institution based | Cross-sectional | General | 412 | >384 | 3.2 | 0.867089765 | | | | | | 0 | | | | | 0 | |
| 17 | Getachew E, etal | 2015 | Not published | Addis Ababa | Institution based | Cross-sectional | General | 520 | >384 | 3.5 | 0.805927559 | Median | 5.6 | 0.7 | 46.9 | 1.722766598 | 1.733943415 | | | | | 0 | |
| 18 | Tefera F, etal | 2015 | 2017 | Amhara | Community based | Cross-sectional | General | 620 | >384 | 11 | 1.256595503 | Not specified | 11.1 | 5 | 24.8 | 2.406945108 | 1.135132421 | | | | | 0 | |
| 19 | Abamech F, etal | 2017 | 2019 | SNNPR | Institution based | Cross-sectional | General | 402 | >384 | 9.2 | 1.441530419 | | | | | | 0 | | | | | 0 | |
| 20 | Gebreegziabher M, etal | 2014 | 2016 | Tigrai | Institution based | Cross-sectional | Health workers | 225 | <384 | 10.7 | 2.060754986 | Mean | 2.4 | 0.43 | 13.29 | 0.875468737 | 0.914817402 | 3.42 | 1.08 | 10.85 | 1.229640551 | 0.797372429 | |
| 21 | Geremew AB, etal | 2017 | 2018 | Amhara | Community based | Cross-sectional | General | 1137 | >384 | 3 | 0.505901844 | Mean | 3.7 | 0.4 | 27 | 1.30833282 | 1.315693567 | 9 | 1.5 | 18 | 2.197224577 | 1.036229388 | |
| 22 | Bayu H, etal | 2015 | 2016 | Tigrai | Community based | Cross-sectional | General | 1186 | >384 | 19.8 | 1.157117428 | Not specified | 2.36 | 1.16 | 4.8 | 0.858661619 | 0.486703674 | | | | | 0 | |
| 23 | Teame H, etal | 2018 | 2019 | Tigrai | Institution based | Case-control | General | 624 | >384 | | | Not specified | 2.42 | 1.22 | 4.77 | 0.88376754 | 0.480649074 | 15.1 | 8.01 | 28.44 | 2.714694744 | 1.153049937 | |
| 24 | Abdulkadir IR, etal | 2013 | Not published | Addis Ababa | Institution based | Cross-sectional | Students | 387 | >384 | 5.2 | 1.128626798 | | | | | | 0 | | | | | 0 | |
| 25 | Solomon K, etal | 2018 | 2019 | Oromia | Institution based | Cross-sectional | HIV Positive | 475 | >384 | 24.8 | 1.981472074 | | | | | | 0 | | | | | 0 | |
| 26 | Emru K, etal | 2015 | Not published | Addis Ababa | Institution based | Cross-sectional | HIV Positive | 411 | >384 | 25.5 | 2.14994483 | | | | | | 0 | | | | | 0 | |
| 27 | Deresse M, etal | 2017 | 2018 | SNNPR | Community based | Cross-sectional | General | 821 | >384 | 15.1 | 1.249599205 | | | | | | 0 | | | | | 0 | |
| 28 | Belete N, etal | 2014 | 2015 | Addis Ababa | Institution based | Cross-sectional | HIV Positive | 322 | <384 | 11.5 | 1.777839781 | | | | | | 0 | | | | | 0 | |
| 29 | Nigussie T, etal | 2017 | 2019 | Oromia | Community based | Cross-sectional | General | 737 | >384 | 15.5 | 1.333093036 | Not specified | 3.47 | 1.47 | 8.21 | 1.244154594 | 0.662283418 | | | | | 0 | |
| 30 | Kassa RT, etal | 2018 | 2019 | Oromia | Community based | Cross-sectional | General | 390 | >384 | 16.1 | 1.861065314 | Mean | 1.2 | 0.7 | 2.4 | 0.182321557 | 0.332612368 | 1.8 | 0.4 | 1.5 | 0.587786665 | 0.267553278 | |
| 31 | Shiferaw S, etal | 2016 | 2018 | Addis Ababa | Institution based | Cross-sectional | HIV Positive | 581 | >384 | 10.8 | 1.287674925 | | | | | | 0 | | | | | 0 | |
| 32 | Bante SA, etal | 2017 | 2019 | Amhara | Community based | Cross-sectional | General | 517 | >384 | 20.9 | 1.788200094 | Median | 0.59 | 0.27 | 1.34 | -0.527632742 | 0.263879603 | 3.38 | 1.92 | 7.61 | 1.217875709 | 0.608513288 | |
| 33 | Tekle T, etal | 2017 | 2020 | SNNPR | Institution based | Cross-sectional | General | 516 | >384 | 22.9 | 1.849779999 | Mean | 0.78 | 0.4 | 1.5 | -0.248461359 | 0.267553278 | | | | | 0 | |
| 34 | Tilahun T etal | 2017 | 2019 | Oromia | Institution based | Cross-sectional | Students | 805 | >384 | 0.5 | 0.248598556 | | | | | | 0 | | | | | 0 | Zero event |
| 35 | Seyoum T, etal | 2017 | 2017 | SNNPR | Institution based | Cross-sectional | Health workers | 281 | <384 | 9.6 | 1.757384015 | Not specified | 1.78 | 1.05 | 3.02 | 0.576613364 | 0.358052777 | | | | | 0 | |
| 36 | Getahun T, etal | 2017 | 2020 | Amhara | Community based | Cross-sectional | General | 821 | >384 | 2.9 | 0.5856485 | | | | | | 0 | | | | | 0 | |
| 37 | Berhanu T, etal | 2018 | 2019 | Addis Ababa | Community based | Cross-sectional | Health workers | 291 | <384 | 9.3 | 1.702545093 | Mean | 1.31 | 0.66 | 2.62 | 0.270027137 | 0.357142857 | 1.13 | 0.57 | 2.26 | 0.122217633 | 0.331632653 | |
| 38 | Gebru Z, etal | 2015 | 2016 | SNNPR | Community based | Cross-sectional | General | 643 | >384 | 5.9 | 0.929213115 | | | | | | 0 | | | | | | |
| 39 | Mulatu K, etal | 2016 | 2017 | SNNPR | institution based | Cross-sectional | Students | 209 | >384 | 14.8 | 2.456277964 | | | | | | 0 | | | | | | |
| 40 | Gelibo T, etal | 2015 | 2017 | National | Community based | Cross-sectional | General | 5823 | >384 | 2.9 | 0.219905058 | | | | | | 0 | | | | | | |
| 41 | Heyi WD, etal | 2016 | 2018 | Oromia | Community based | Cross-sectional | General | 845 | >384 | 5.8 | 0.80410191 | Mean | 6.95 | 2.59 | 18.57 | 1.93874166 | 1.019770209 | | | | **0.399638745** | **0.443251356** | |
| 42 | Aweke YH, etal | 2015 | 2017 | SNNPR | Community based | Cross-sectional | General | 583 | >384 | 9.9 | 1.236931688 | Mean | | | | | | | | | | 0 | |
| 43 | Tadesse A, etal | 2014 | Not published | Oromia | Institution based | Cross-sectional | Students | 667 | >384 | 2.2 | 0.567960131 | Not specified | | | | **4.543859649** | **0.68752508** | | | | | | Using abcand d |
| 44 | Andargie A, etal | 2013 | 2015 | Amhara | institution based | Cross-sectional | General | 603 | >384 | 8.3 | 1.123479256 | Not specified | | | | **4.414141414** | **0.34217498** | | | | **8.444575472** | **0.395549057** | |
